# Towards Antiviral shRNAs Based on the AgoshRNA Design

**DOI:** 10.1371/journal.pone.0128618

**Published:** 2015-06-18

**Authors:** Ying Poi Liu, Margarete Karg, Elena Herrera-Carrillo, Ben Berkhout

**Affiliations:** Laboratory of Experimental Virology, Department of Medical Microbiology, Center for Infection and Immunity Amsterdam (CINIMA), Academic Medical Center, University of Amsterdam, Meibergdreef 15, 1105 AZ, Amsterdam, the Netherlands; Wuhan University, CHINA

## Abstract

RNA interference (RNAi) can be induced by intracellular expression of a short hairpin RNA (shRNA). Processing of the shRNA requires the RNaseIII-like Dicer enzyme to remove the loop and to release the biologically active small interfering RNA (siRNA). Dicer is also involved in microRNA (miRNA) processing to liberate the mature miRNA duplex, but recent studies indicate that miR-451 is not processed by Dicer. Instead, this miRNA is processed by the Argonaute 2 (Ago2) protein, which also executes the subsequent cleavage of a complementary mRNA target. Interestingly, shRNAs that structurally resemble miR-451 can also be processed by Ago2 instead of Dicer. The key determinant of these “AgoshRNA” molecules is a relatively short basepaired stem, which avoids Dicer recognition and consequently allows alternative processing by Ago2. AgoshRNA processing yields a single active RNA strand, whereas standard shRNAs produce a duplex with guide and passenger strands and the latter may cause adverse off-target effects. In this study, we converted previously tested active anti-HIV-1 shRNA molecules into AgoshRNA. We tested several designs that could potentially improve AgoshRNA activity, including extension of the complementarity between the guide strand and the mRNA target and reduction of the thermodynamic stability of the hairpins. We demonstrate that active AgoshRNAs can be generated. However, the RNAi activity is reduced compared to the matching shRNAs. Despite reduced RNAi activity, comparison of an active AgoshRNA and the matching shRNA in a sensitive cell toxicity assay revealed that the AgoshRNA is much less toxic.

## Introduction

Short hairpin RNAs (shRNAs) can be expressed intracellularly from transgenes and have been widely used to silence specific genes by induction of the RNAi pathway. RNAi is an evolutionary conserved gene silencing mechanism that is triggered by small double-stranded RNAs [1, 2]. Designed shRNAs structurally resemble the large class of cellular miRNAs [3]. The miRNAs are expressed as primary transcript and processed by the nuclear Drosha endonuclease into a pre-miRNA, which is transported to the cytoplasm by Exportin-5 and further processed by the Dicer endonuclease into miRNA duplexes. The miRNA duplex associates with an Argonaute protein in the RNA-induced silencing complex (RISC), resulting in cleavage and removal of the passenger strand and subsequent annealing of the guide strand to a complementary target mRNA, causing its inactivation by translational suppression or cleavage-mediated inactivation. Unlike miRNAs, the man-made shRNAs are produced as small transcripts that do not require Drosha processing, but are recognized directly by Dicer to produce small interfering RNAs (siRNAs) that are fully complementary to a specific mRNA. More recently, some notable exceptions to the standard miRNA processing pathway were described. Non-canonical miRNA processing routes are used by the so-called miRtrons [4–6], tRNAZ [7, 8] and small nucleolar RNAs [9–12] that do not require Drosha, but these miRNAs remain dependent on Dicer for their maturation. Recently, miR-451 was described to use Ago2 instead of Dicer for its maturation [13–15]. Likewise, shRNAs that are independent of Dicer and dependent on Ago2 for processing have been reported [16–20]. These Dicer-independent miRNA and shRNA molecules are characterized by a relatively short basepaired stem, which likely prevents Dicer recognition. We termed this shRNA subclass “AgoshRNAs” because of the dual dependence on Ago2 for maturation in addition to regular mRNA silencing.

**Fig 1:**
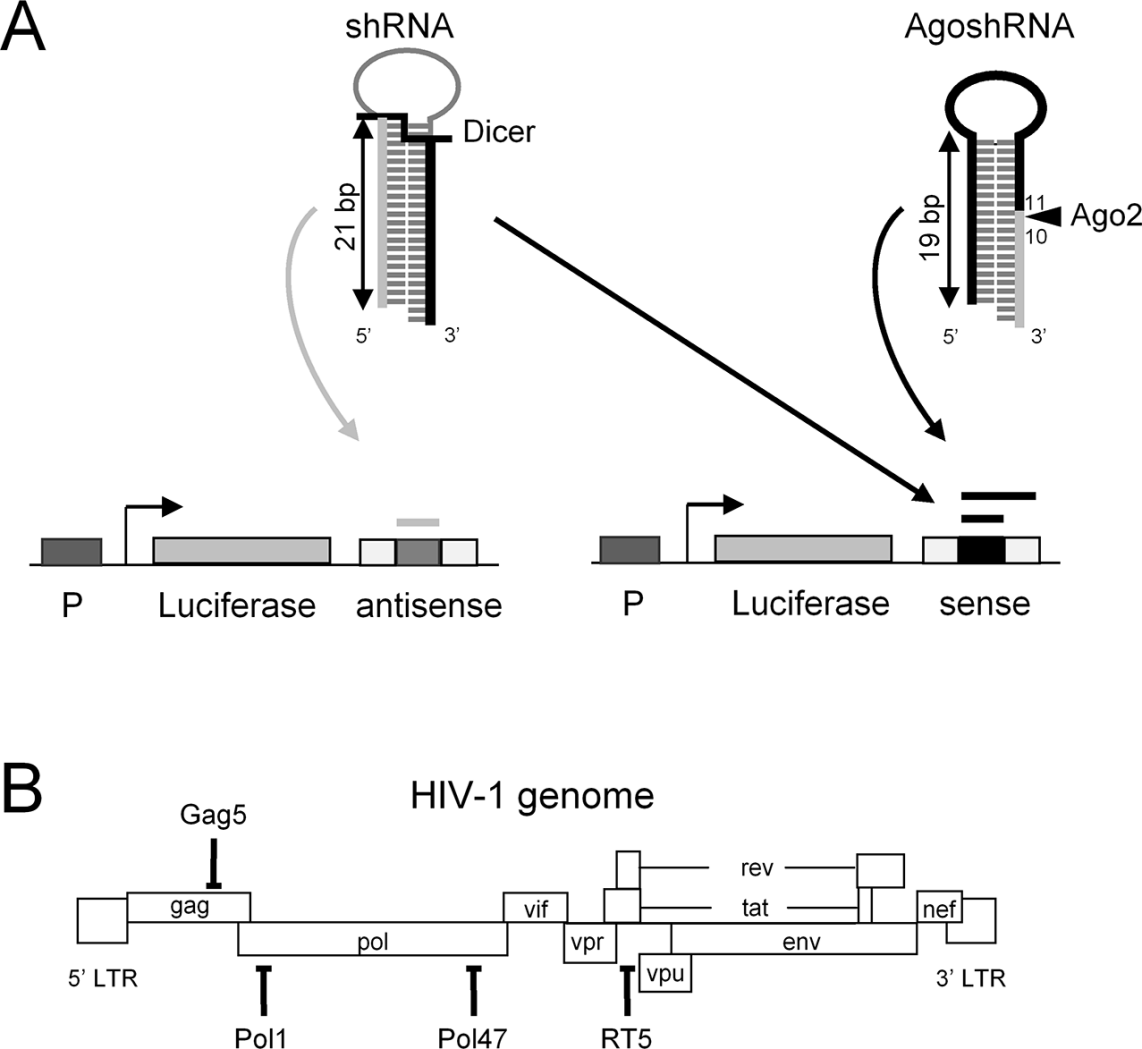
Anti-HIV shRNAs and AgoshRNAs. **(A)** Canonical shRNA processing by Dicer (**¯**I**_**) and non-canonical AgoshRNA processing by Ago2 (**◄**). The guide strand is marked black and bold. Luciferase reporters encoding the HIV-sense or HIV-antisense target sequence. HIV-sense scores the activity of the shRNA guide strand and the AgoshRNA guide strand. The HIV-antisense reporter scores the activity of the shRNA passenger strand (marked in grey). **(B)** Indicated are four target sequences (Gag5, Pol1, Pol47 and RT5) in the HIV-1 genome with the names of the (Ago)shRNA inhibitors.

AgoshRNA processing by Ago2 differs significantly from regular shRNA processing by Dicer, thus yielding quite different RNA products. Ago2 creates a single-strand cut halfway the duplex on the 3’ side, whereas Dicer generates a double-strand break near the top of the duplex (Fig 1A). Consequently, AgoshRNA processing yields a single RNA product of ~30 nucleotides (nt) as active species, whereas a shRNA is converted into a regular siRNA duplex, of which both the guide and passenger strand are theoretically active in RNAi-silencing. Thus, the AgoshRNA design has the clear advantage of lacking potential off-target effects caused by the passenger strand. We previously listed other advantages of AgoshRNA inhibitors [20], including their ability to remain active in cells such as monocytes that express no or hardly any Dicer [21]. The AgoshRNA design also has some potential disadvantages. For instance, the 3’ extension of the active strand (Fig 1A) may cause a steric clash with the Ago2 enzyme, consistent with the observation that large hairpin loops hinder AgoshRNA activity [13–16]. We previously described that the hairpin duplex length is the most critical parameter for shRNA versus AgoshRNA processing, with hairpins around 18 bp being too small for Dicer and ideally suited for Ago2 processing [16, 22]. In this study, we designed and tested several AgoshRNAs against highly conserved sequences of the HIV-1 RNA genome and compared their activity with regular shRNAs that target the same HIV-1 sequences. We specifically tested whether the AgoshRNA architecture with an extended ~30 nt guide strand allows one to extend the basepairing complementarity with the target HIV-1 RNA by changing the loop sequence. Such a manipulation is impossible for regular shRNA reagents because the loop is removed by Dicer. A processed AgoshRNA strand can still fold the upper half of the original hairpin (Fig 1A), which may hinder target RNA annealing. We therefore tested whether the introduction of weak G-U bp along the AgoshRNA stem could boost the silencing activity. The findings of this study can aid the future development of active and safe AgoshRNA-based therapeutics.

## Materials and Methods

### Plasmid construction

For the AgoshRNA constructs, complementary DNA oligonucleotides encoding the AgoshRNA sequence with BamHI and HindIII sites were annealed and inserted into corresponding restriction sites of the pSUPER vector [1]. All hairpin RNA constructs were sequence-verified using the BigDye Terminator Cycle Sequencing kit (ABI, Foster City, CA, USA). For sequencing of hairpin RNA constructs a sample denaturation temperature of 98°C was used and 1M Betaine was included in the reaction mixture. The Mfold program was used to predict the secondary structure and thermodynamic stability of the AgoshRNA transcripts [23].

### Cell culture

Human embryonic kidney 293T (ATCC CRL-11268) and HCT-116 (ATCC CCL-247) adherent cells were grown as monolayer in Dulbecco’s modified Eagle’s medium (Life Technologies, Invitrogen, Carlsbad, CA) supplemented with 10% fetal calf serum (FCS), penicillin (100 U/ml), streptomycin (100 μg/ml) and minimal essential medium non-essential amino acids (DMEM/10% FCS) in a humidified chamber at 37°C and 5% CO_2_. SupT1 T cells were grown in Advanced RPMI (Gibco BRL, Carlsbad, CA) supplemented with L-glutamine, 1% FCS, penicillin (30 U/ml) and streptomycin (30 μg/ml) in a humidified chamber at 37°C and 5% CO_2_.

### Luciferase assays

HEK293T cells were seeded one day before transfection in 24-wells plates at a density of 1.4 x 10^5^ cells/well in 500 μl DMEM/10% FCS without antibiotics. The cells were co-transfected with 100 ng Firefly luciferase reporter construct and 2.5, 10 or 40 ng of AgoshRNA construct using Lipofectamine 2000 reagent according to the manufacturer’s protocol. To normalise for cell viability and transfection efficiency, 1 ng of pRL plasmid (Promega) expressing renilla luciferase from the CMV promoter was included. We added pBluescript SK- (pBS) (Promega) to obtain equal DNA concentrations. Two days post-transfection, firefly and renilla luciferase expression was measured using the Dual-Luciferase Reporter Assay System (Promega) according to the manufacturer’s instructions. Relative luciferase activities were calculated from the ratio between firefly and renilla luciferase expression. We performed three independent transfections, each in duplicate. Values were corrected for between-session variation as described previously [24]. The resulting six values were used to calculate the standard deviation shown as error bar.

### Lentiviral vector production and transduction

The lentiviral vector was produced and titrated as described previously [25, 26]. Lentiviral vector plasmids encoding the hairpins are derived from the construct JS1 (pRRLcpptpgkgfppreSsin) [27]. The vector was produced by co-transfection of lentiviral vector plasmid and packaging plasmids pSYNGP [28], pRSV-rev and pVSV-g [29] with Lipofectamine 2000 (Invitrogen, Carlsbad, CA). After transfection, the medium was replaced with OptiMEM (Invitrogen, Carlsbad, CA). The lentiviral vector containing supernatant was collected, filtered (0.45 μm) and aliquots were stored at −80°C. The transduction titer was measured via GFP expression. SupT1 cells were transduced at a multiplicity of infection (moi) of 0.15. Three days after transduction, live cells were selected by fluorescence-activated cell sorting (FACS) for green fluorescent protein expression.

### HIV-1 inhibition assays

HEK293T cells were seeded one day before transfection in 24-wells plates at a density of 1.4 x 10^5^ cells/well in 500 μl DMEM/10% FCS without antibiotics. The cells were co-transfected with 250 ng of the full-length HIV-1 molecular clone pLAI, 1 ng of pRL-CMV and 2.5, 10 or 40 ng of AgoshRNA construct using Lipofectamine 2000. We added pBS to have an equal DNA concentration per transfection. Two days post-transfection, virus production was determined by measuring the CA-p24 levels in the culture supernatant by ELISA as reported previously [30]. Cells were lysed and the lysates used to measure the renilla luciferase activities with the Renilla Luciferase Assay System (Promega) according to the manufacturer’s protocol. Relative HIV-1 production was calculated as the ratio between the CA-p24 level and the renilla luciferase activity. Values were corrected for between-session variation as described previously [24].

Transduced SupT1 T cells (1 × 10^6^ cells in 5 ml medium) were challenged with HIV-1 LAI at an moi of 0.02. Virus spread was monitored by measuring CA-p24 production and scoring of syncytia formation every 2 days. Cells were passaged twice a week.

### Sequencing proviral target regions

Cellular DNA of the infected cell with the integrated provirus was isolated as previously described [31]. Integrated proviral DNA sequences were PCR amplified with the following primer pairs: sense (CAGACCATCAATGAGGAAGCTGCAGAATGGGAT; position 1445) and antisense (CCCTGGCCTTCCCTTGTAGGAAAACCAGATCTTCCC; position 2141) with 30 cycles (1 minute of denaturation at 96°C, 1 minute of annealing at 62°C, and 2 minutes of extension at 72°C). The PCR products were sequenced with the Big Dye Terminator Cycle Sequencing kit (ABI) using the same primers.

### Northern blot analyses

For siRNA analyses, 1.5 x 10^6^ HCT-116 cells were seeded in T25 flasks in 4 ml of DMEM/10% FCS without antibiotics. The cells were transfected with 5 μg AgoshRNA or shRNA construct using Lipofectamine 2000 reagent. Two days post-transfection, small RNAs were extracted from transfected HCT-116 cells using the mirVana miRNA isolation kit (Life Technologies, Ambion, Austin, TX) according to the manufacturer’s protocol. RNA concentrations were determined on the Nanodrop 1000 (Thermo Fisher Scientific). 15 μg of the total RNA was resolved on a 15% denaturing polyacrylamide gel containing urea (Life Technologies). We used the Decade RNA molecular weight marker (Life Technologies) alongside the cellular RNA. To ensure equal sample loading, ribosomal RNA bands were visualised by staining the gels with 2 μg/ml ethidium bromide and subsequent exposure to UV light. The RNA was electrotransferred to a positively charged nylon membrane (Boehringer Mannheim, GmbH, Mannheim, Germany) according to the manufacturer’s instructions. After blotting, the RNA was cross-linked to the membrane using UV light at a wavelength of 254 nm (1200 μJ x 100). The membranes were hybridized overnight at 42°C with locked nucleic acid (LNA) oligonucleotides in 10 ml ULTRAhyb hybridization buffer (Life Technologies, Austin, TX). LNA oligonucleotide probes were 5’-end labelled with the kinaseMax kit (Life Technologies) in the presence of 1 μl [γ-32P] ATP (0.37 MBq/μl, Perkin Elmer). The probes were purified on Sephadex G-25 columns (Amersham Biosciences) to remove unincorporated nucleotides according to the manufacturer’s protocol. We used the following oligonucleotides to detect the antisense (5’) strand of the siRNA (LNA-positions are underlined): Pol47: 5′-GTGAAGGGGCAGTAGTAAT-3′, Pol1: 5’-ACAGGAGCAGATGATACAG-3’, Gag5 probe: 5’- GAAGAAATGATGACAGCAT-3’ and RT5 probe: 5’-ATGGCAGGAAGAAGCGGAG-3’. To detect the (3’) sense strand of the siRNA the following oligonucleotides were used (LNA positions are underlined): Pol47: 5’-ATTACTACTGCCCCTTCAC-‘3, Pol1: 5’-CTGTATCATCTGCTCCTGT-3’, Gag5: 5’-ATGCTGTCATCATTTCTTC-3’, RT5: 5’-CTCCGCTTCTTCCTGCCAT-3’.

### Competitive cell growth assay

Lentivirally transduced SupT1 T cells were generated using an moi of 0.15. Four days after infection, cells were sorted for GFP expression by fluorescence-activated cell sorted. Transduced SupT1 cells were screened for a negative impact on cell growth as induced by lentiviral integration and/or shRNA expression using the competitive cell growth assay. In brief, transduced SupT1 cells (GFP+/shRNA+) were mixed with approximately 80% untransduced cells (GFP-). The GFP+/- ratio was analysed over a 50-day period of co-culture by fluorescence-activated cell sorting. The impact on cell growth was converted as percentage reduction in cell growth [32].

## Results

### Design of four anti-HIV AgoshRNAs

We previously selected four potent anti-HIV shRNAs in a large screen against highly conserved sequences in viral RNA genome: Gag5, Pol1, Pol47, and RT5 (Fig 1B). The guide strand is generated from the 3’ side of these regular shRNAs (Fig 1A, left). We now designed the four matching AgoshRNAs with the same guide strand sequence, but now positioned on the 5’ side of the hairpin (Fig 1A, right). The shRNA is processed by Dicer that cleaves off the loop, whereas the AgoshRNA is processed by Ago2, which generates a single strand RNA break on the 3’ side between the 10^th^ and 11^th^ bp. The activity of these four hairpins was tested on matching luciferase reporter constructs with either the sense HIV-1 target or the antisense sequence. The HIV-sense reporter detects the activity of the shRNA guide strand (black thick line) and HIV-antisense will score any passenger strand activity (grey thick line). The AgoshRNA design should yield a single, extended guide strand from the 5’ side (marked as thick line), which is scored on the HIV-sense luciferase reporter.

**Fig 2:**
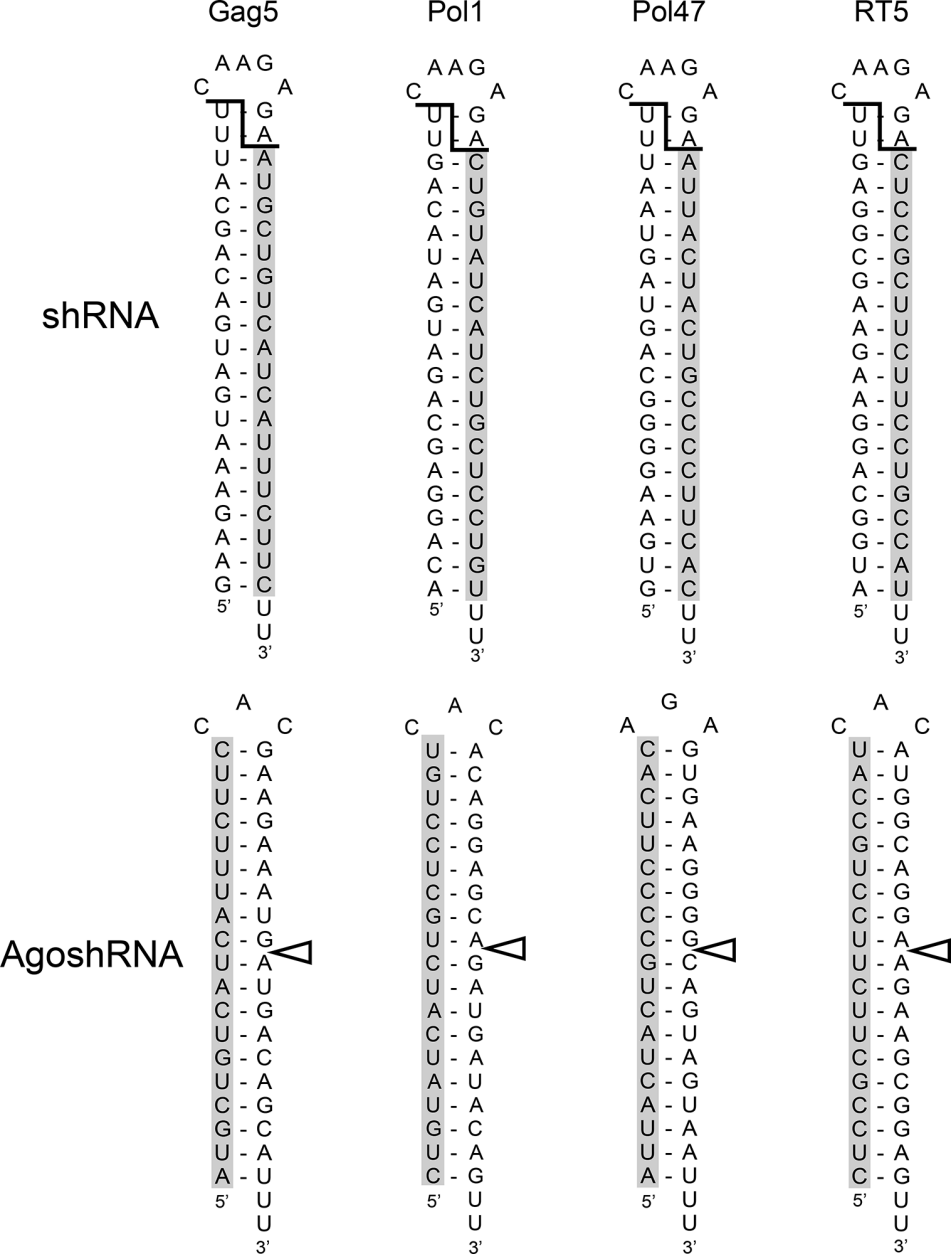
Four potent anti-HIV shRNAs and the matching AgoshRNAs. The four shRNAs shown are potent antivirals that target highly conserved HIV-1 RNA sequences. The Dicer processing sites are indicated (**¯**I**_**), with the 3’-guide strand highlighted in grey (**◄**). Based on these guide strands, four matching Aggo2 molecules were designed against the same HIV-1 targets. The Ago2 processing site is indicated and the 5’-guide is highlighted in grey.

**Fig 3:**
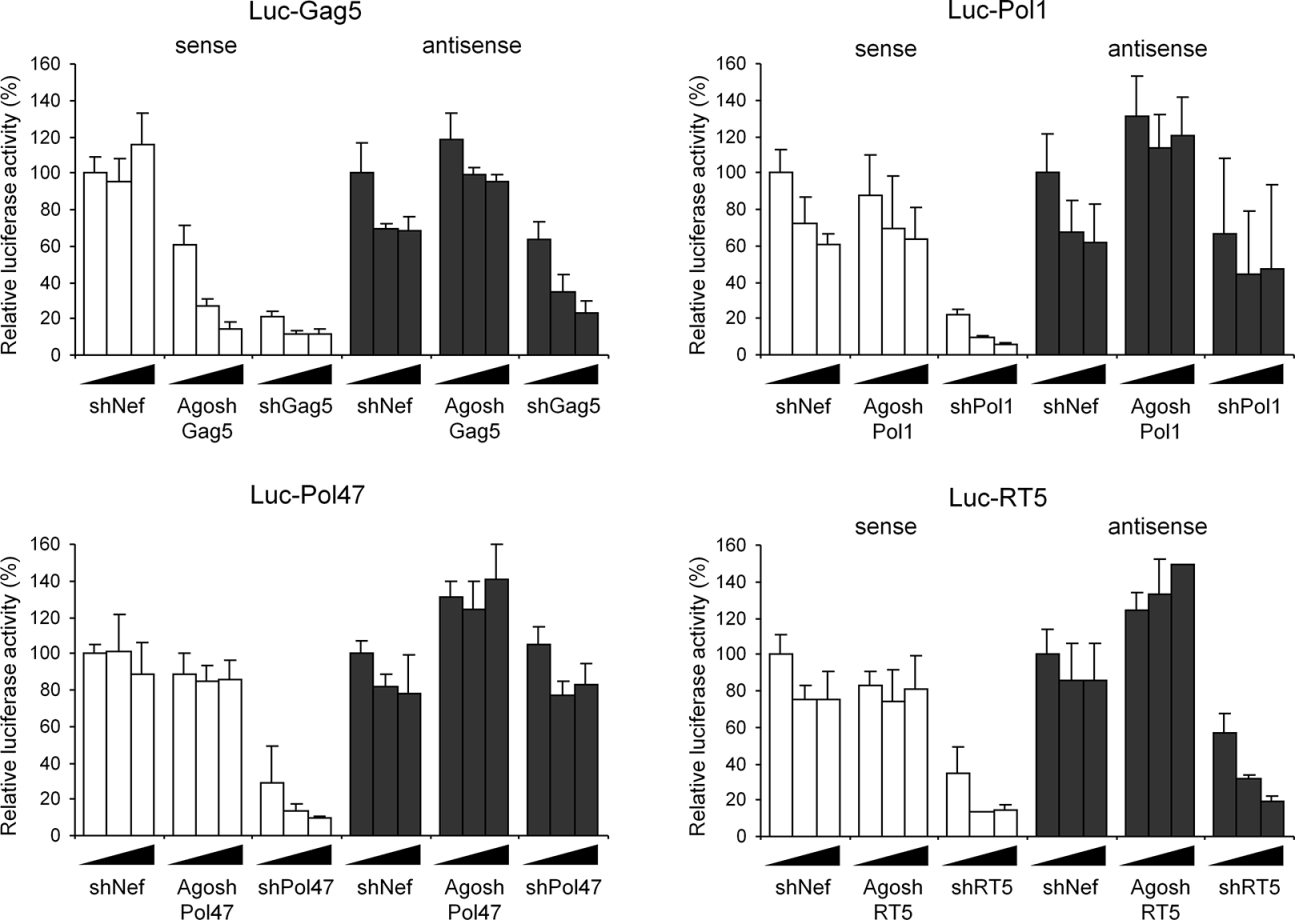
Knockdown activity of the 5’/ 3’ strands of the (Ago)shRNAs against HIV-1. Luciferase knockdown by the shRNA 5’-passenger and 3’-guide versus AgoshRNA 5’-guide was determined by co-transfection of a luciferase reporter with one of the four (Ago)shRNA constructs. 293T cells were co-transfected with 100 ng of the respective firefly luciferase reporter plasmid, 1 ng of renilla luciferase plasmid, and 2.5, 10 or 40 ng of the corresponding shRNA constructs. An irrelevant shRNA (shNef) served as negative control, for which the activity was set at 100% luciferase expression. The HIV-sense reporter (white bars) scores shRNA guide activity and AgoshRNA guide activity, whereas the HIV-antisense (dark bars) scores shRNA passenger strand activity. We performed three independent transfections, each in duplicate, and standard deviations were calculated.

The four shRNA and AgoshRNA variants targeting different regions of the HIV-1 genome are depicted in Fig 2. Indicated are the predicted Dicer and Ago2 cleavage sites and the guide strands are marked in grey. The silencing activity was scored in HEK293T cells transfected with a luciferase reporter and an increasing amount of the matching hairpin construct (Fig 3). The luciferase activity measured in the presence of the unrelated shNef construct was set at 100%. As expected, all four previously selected shRNAs produce active guide strands that target the corresponding HIV-sense reporter, but considerable passenger strand activity was scored for shGag5 and shRT5 on the matching HIV-antisense reporter. Of the four designed AgoshRNAs, potent luciferase knock down was observed only for AgoshGag5 on the matching HIV-sense reporter. Importantly, no passenger strand activity was measured on the HIV-antisense reporter, as predicted for the AgoshRNA design. The other AgoshPol1, AgoshPol47 and AgoshRT5 molecules did not show any inhibitory potential on either reporter. Although this initial result may seem disappointing, we should stress that the four potent shRNAs were selected in a large screen of 86 shRNA candidates, of which the majority were also marginally active or inactive [26].

### Extending the target complementarity in the AgoshRNA design

**Fig 4:**
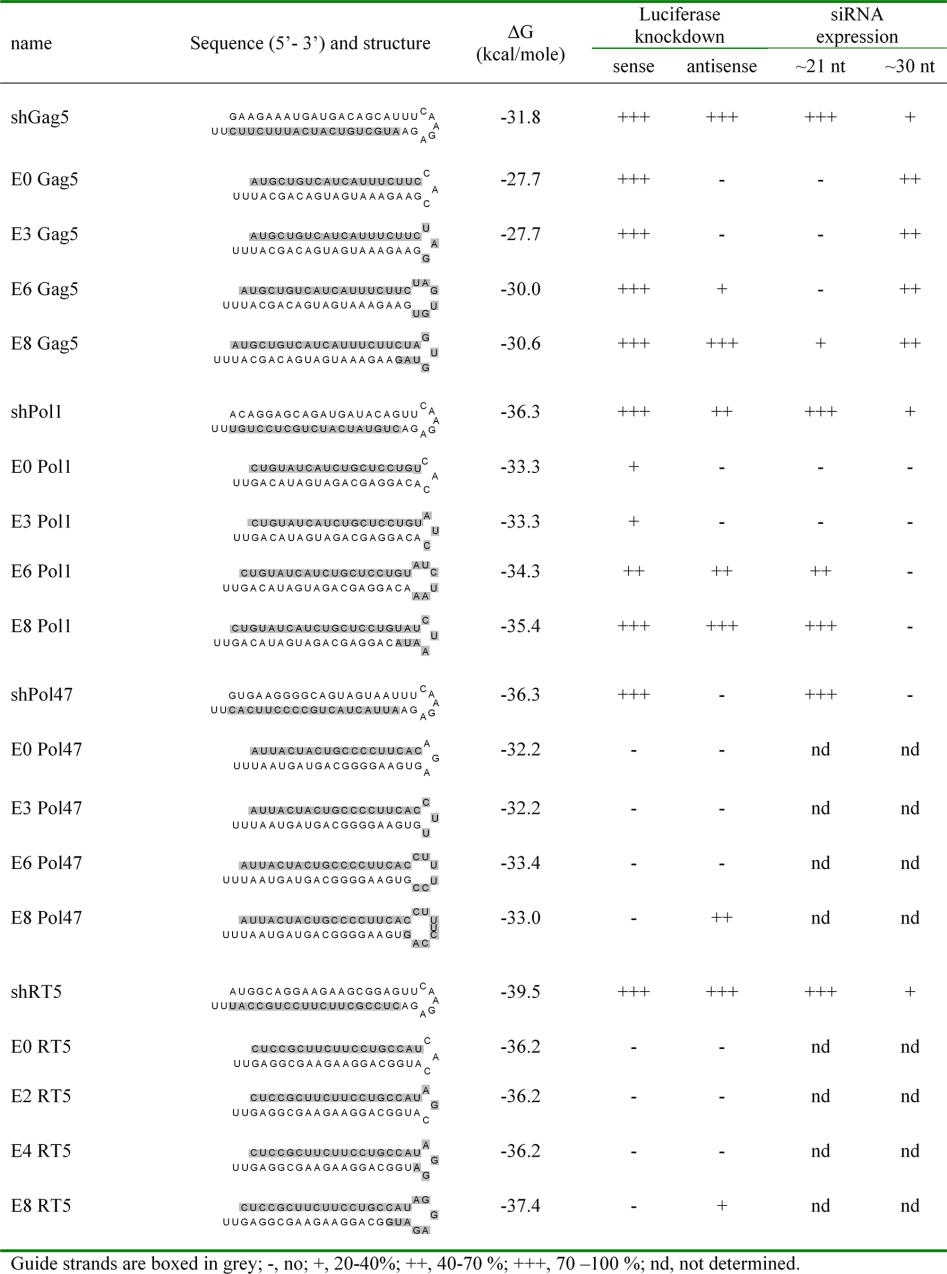
Structure and properties of the anti-HIV AgoshRNAs.

**Fig 5:**
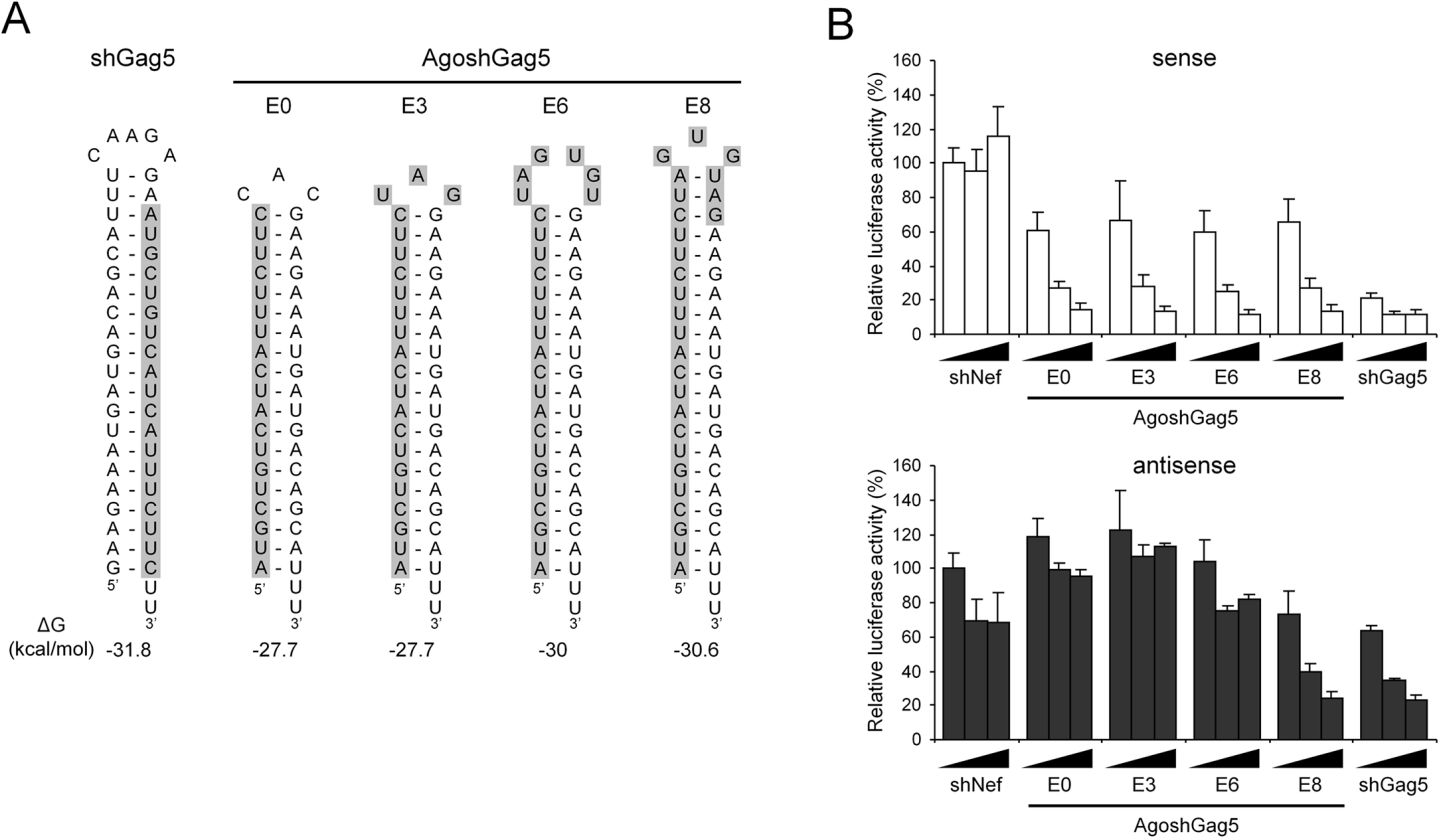
Design of AgoshRNAs with an extended guide. **(A)** The 19-nt guide strand of the original shGag5 and the derived AgoshGag5 are boxed in grey. The complementarity between the guide and the HIV-1 target sequence was increased in variants E3, E6 and E8 by addition of anti-HIV nt in the loop and into the 3’ strand of the stem. The thermodynamic stability (ΔG in kcal/mole) was determined using the Mfold program. **(B)** Knockdown activity on the HIV-sense and HIV-antisense reporters (upper and lower graph, respectively). See Fig 3 for further details.

Unlike the shRNA design, AgoshRNAs generate an extended guide strand, of which only 19 nt are designed to be complementary to the HIV-1 target. We therefore tested whether the activity can be boosted by extension of this complementarity by adaptation of the adjacent sequences that form the hairpin loop and the upper 3’ stem side. We tested whether extended guide strands could enhance the activity of AgoshGag5 and create active versions of the three inactive molecules AgoshPol1, AgoshPol47 and AgoshRT5. The structure and thermodynamic stability of all hairpin constructs that is the original shRNA and four AgoshRNA variants, are listed in Fig 4 for each inhibitor (Gag5, Pol1, Pol47 and RT5). The new hairpin designs were named according to the number of additional HIV-complementary nt added on the 3’ side. For instance, the original AgoshGag5 variant has a 19-nt guide, to which 3, 6 and 8 additional nt were added to extend its complementarity to the HIV target, yielding the extended variants E3, E6 and E8 variants of the original AgoshGag5 design, which was named E0 (Fig 5A, guide strand marked in grey). Note that these mutations may affect the structure near the top of the hairpin. More specifically, a larger hairpin loop is predicted for the E6 variant and two additional bp are predicted for the E8 variant (Fig 5A).

All AgoshGag5 variants demonstrated knockdown activity on the HIV-sense reporter, but the activity was not boosted by extension of the target complementarity (Fig 5B, upper graph). We used the unrelated shNef as negative control and shGag5 as highly active positive control. Knockdown activity on the HIV-antisense reporter was absent for all AgoshRNA variants, except for the E8 mutant (Fig 5B, lower graph). In fact, the AgoshRNA variant E8 has likely become a Dicer template due to the additional 2 bp, creating an extended 21 bp stem that is ideal for Dicer recognition. According to this scenario, E8 is processed into siRNA duplexes with guide and passenger activity. Indeed, this variant showed inhibitory activity on both the sense and the antisense luciferase reporters, similar to the Dicer-processed shGag5 control. Such adverse activity was not scored for the 19 bp AgoshRNAs that are alternatively processed by Ago2 in a single active guide strand.

The inhibitory activities of all shRNA and AgoshRNA hairpins are summarized in Fig 4. For the AgoshPol1 set of inhibitors, the original version E0 and variant E3 showed moderate (20–40%) inhibition of the HIV-sense reporter, which was boosted to 70–100% inhibition for the E6 and E8 variants. However, the latter two constructs equally affected the HIV-antisense reporter, suggesting perhaps that these hairpins are processed by Dicer into dually-active siRNAs. This may be expected for E8 variant because the duplex has become 21 bp, but no simple explanation is available for the E6 variant. Overall, extension of the guide strand complementarity “over the loop” did not increase the inhibitory potential of these AgoshRNAs.

### Processing of the different shRNA/AgoshRNA constructs

**Fig 6:**
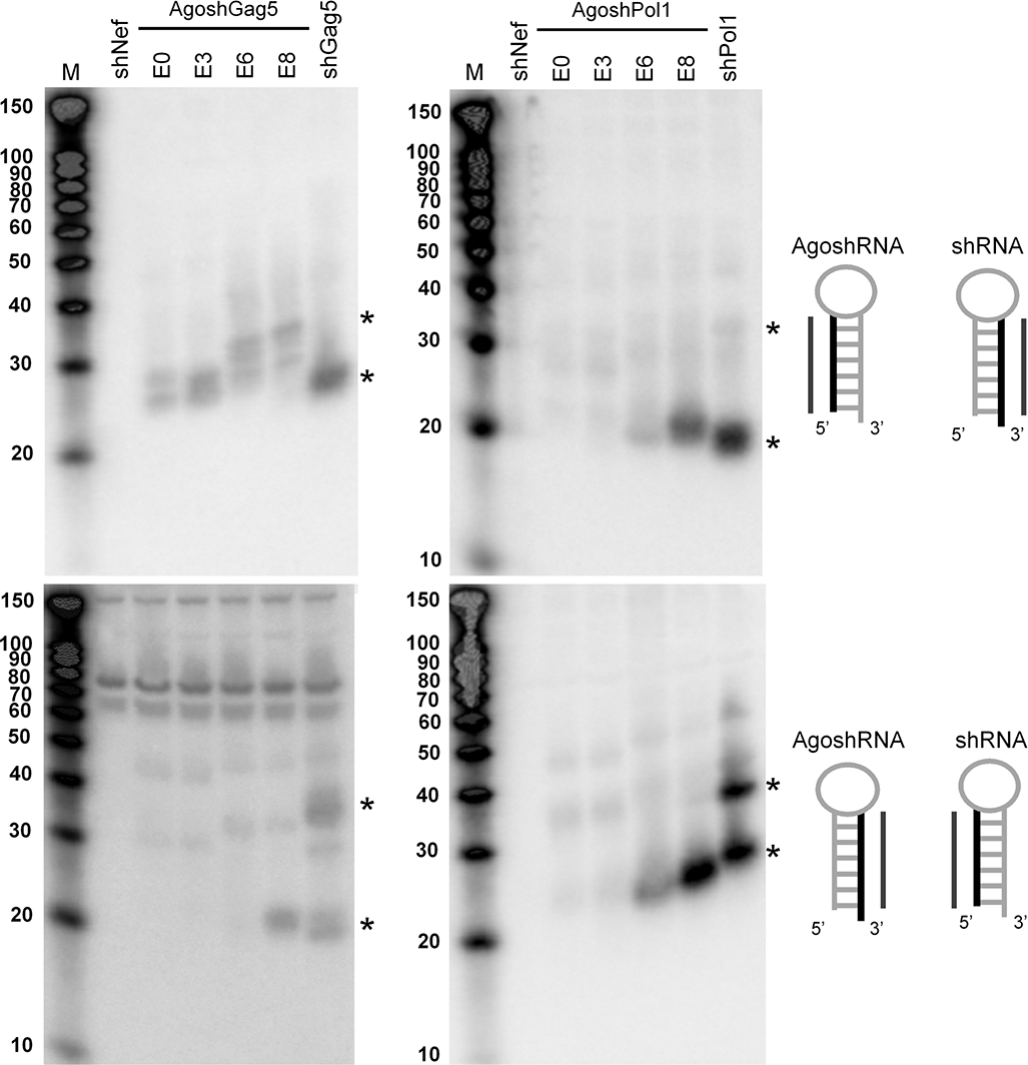
Northern blotting of the (Ago)shRNA processing products. Processing of the 5’ strand (upper panels) and 3’ strand (lower panels) of AgoshGag5 and AgoshPol1 variants was analyzed by Northern blot analysis. The asterisks indicate the regular Dicer processed small RNAs of 21 nt and the Ago2 products of ~33 nt. The RNA structure cartoon indicates which side of the hairpin was probed for AgoshRNAs and the control shRNAs.

We thus far scored a wide range of silencing activities for the different anti-HIV AgoshRNAs. This could truly reflect altered RNAi activity, but could also be due to differences in intracellular processing efficiency, the use of a different pathway (Ago2 versus Dicer) or differences in stability of the mature RNA molecules. Northern blot analyses were performed to analyse the intracellular processing of all AgoshRNA constructs and the shRNA controls. The results obtained for the active AgoshGag5 set and the moderately active AgoshPol1 set are shown in Fig 6. The processing pattern of all shRNA and AgoshRNA hairpins is summarized in Fig 4.

The original AgoshGag5 (E0) construct showed processing products exclusively with the probe that detects the 5’ side of the AgoshRNA and the 3’ side of the matching shRNA (Fig 6, upper left panel). Two bands were visible of ~26 and ~30 nt in length for the original E0 hairpin and the E3 mutant, which could be the predicted guide strand and a 3’-trimmed product (Harwig and Berkhout, unpublished results). Both fragments increased in size for the E6 and E8 variants with 6 and 8 additional complementary nt in or around the hairpin loop. This shift in size is consistent with these products being generated by Ago2 cleavage halfway the 3’ side of the duplex. As previously described, the 10 nt bystander product is not detected on Northern blot analysis [16]. The control shGag5 construct is processed by Dicer into a regular double-stranded siRNA of ~21 bp, which shows up with both probes (Fig 6, left panels). Probing of the 5’ side of shGag5 (lower left panel) also detects a ~33 nt processed RNA, which corresponds to the size of an Ago2-processed product. This phenomenon has been observed before when we used the pSUPER loop UUCAAGAGA, which can form two additional, but relatively weak bp (U-G and U-A). Thus, this structure may exist as a mixture of hairpins of 19, 20 and 21 bp. The shorter variants are subjected to Ago2 mediated processing, while the longer variants will be substrates for Dicer. A similar 21 nt band is observed for the E8 construct (Fig 6, lower left panel). Construct E8 seems to have a hybrid character concerning Ago2 versus Dicer processing, which is due to the increased stem length. Indeed, E8 gained activity on the antisense reporter, without losing activity on the sense reporter.

The original AgoshPol1 construct (E0) showed no pronounced processing products, consistent with its poor activity on either reporter (Fig 6, upper right panel). The same is true for the E3 variant, but E6 and E8 gained some activity on both reporters and yielded characteristic ~21 nt Dicer products with both probes, similar in size to the regular Dicer products of the shPol1 control (Fig 6, right panels). Similar to shGag5, a ~33 nt processed product was detected from the 5’ side of the shPol1 control. This again relates to the hybrid character of these shRNAs. All these signatures (product length, activity on both reporters) are consistent with a switch from Ago2 to Dicer-mediated processing, which is a partial switch for E6 and a complete reversal for E8. For the AgoshPol47 and AgoshRT5 sets we observed no improvement by extending the guide strand with complementary nt. Strangely, both the E8 variant of Pol47 and RT5 showed activity on the antisense reporter and not the sense reporter. This cannot be explained either by Ago2 or Dicer mediated processing.

### Anti-HIV activity of the AgoshRNA molecules

**Fig 7:**
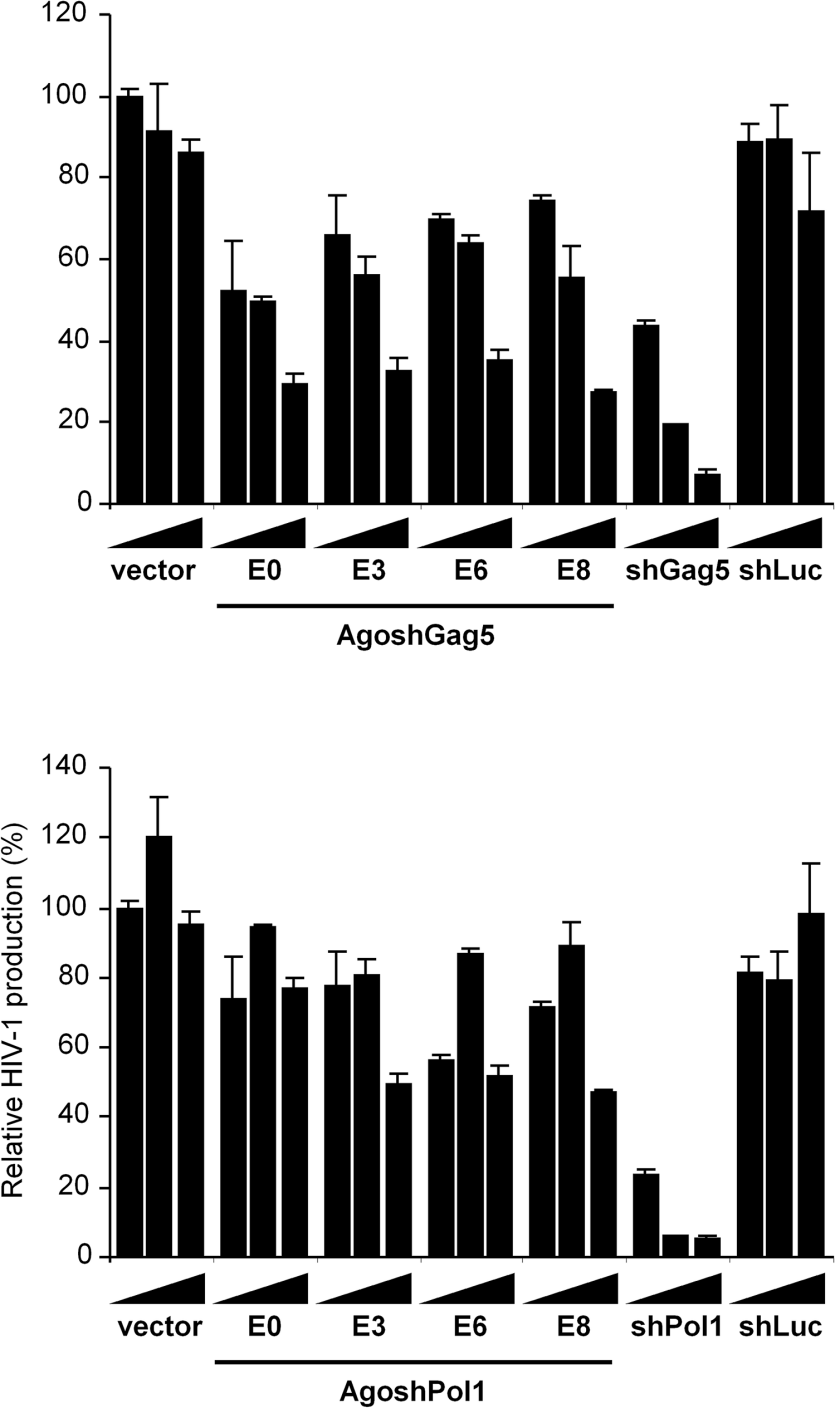
Knockdown activity of the AgoshRNA 5’ guide on HIV-1 production. The knockdown activity of the 5’ strands of the AgoshGag5 (upper graph) and AgoshPol1 variants (lower graph) were tested by co-transfection of 2.5, 10 or 40 ng of AgoshRNA construct together with 250 ng of the full-length HIV-1 molecular clone pLAI and 1 ng of Renilla plasmid. The CA-p24 levels in the supernatant were determined as a measure for HIV-1 production. HIV-1 production in the vector transfected cell culture was set at 100%. We performed three independent transfections, each in duplicate, and standard deviations were calculated.

Thus far, the AgoshRNA molecules and the shRNA controls were tested on matching luciferase reporters, but they all have in common that they target the HIV-1 RNA genome (Fig 1A). To test the ability of the active AgoshGag5 and AgoshPol1 hairpins to inhibit HIV-1 production, we co-transfected different amounts of the hairpin constructs with the HIV-1 molecular clone pLAI into 293T cells and measured viral Gag protein production by CA-p24 Elisa in the culture supernatant. The unrelated shLuc construct was included as negative control. A Renilla luciferase construct was included to allow a correction for transfection variation. We scored similar antiviral activity for the original E0 version of AgoshGag5 and the three E variants, but clearly less than the positive control shGag5 (Fig 7, upper panel). These results are consistent with the luciferase knockdown experiments (Figs 3 and 5B). All AgoshPol1 designs showed very little HIV-1 knockdown when compared to the positive control shPol1 (Fig 7, lower panel). In sum, these results indicate that extension of the AgoshRNA guide strand “over the loop” does not improve the inhibitory capacity.

**Fig 8:**
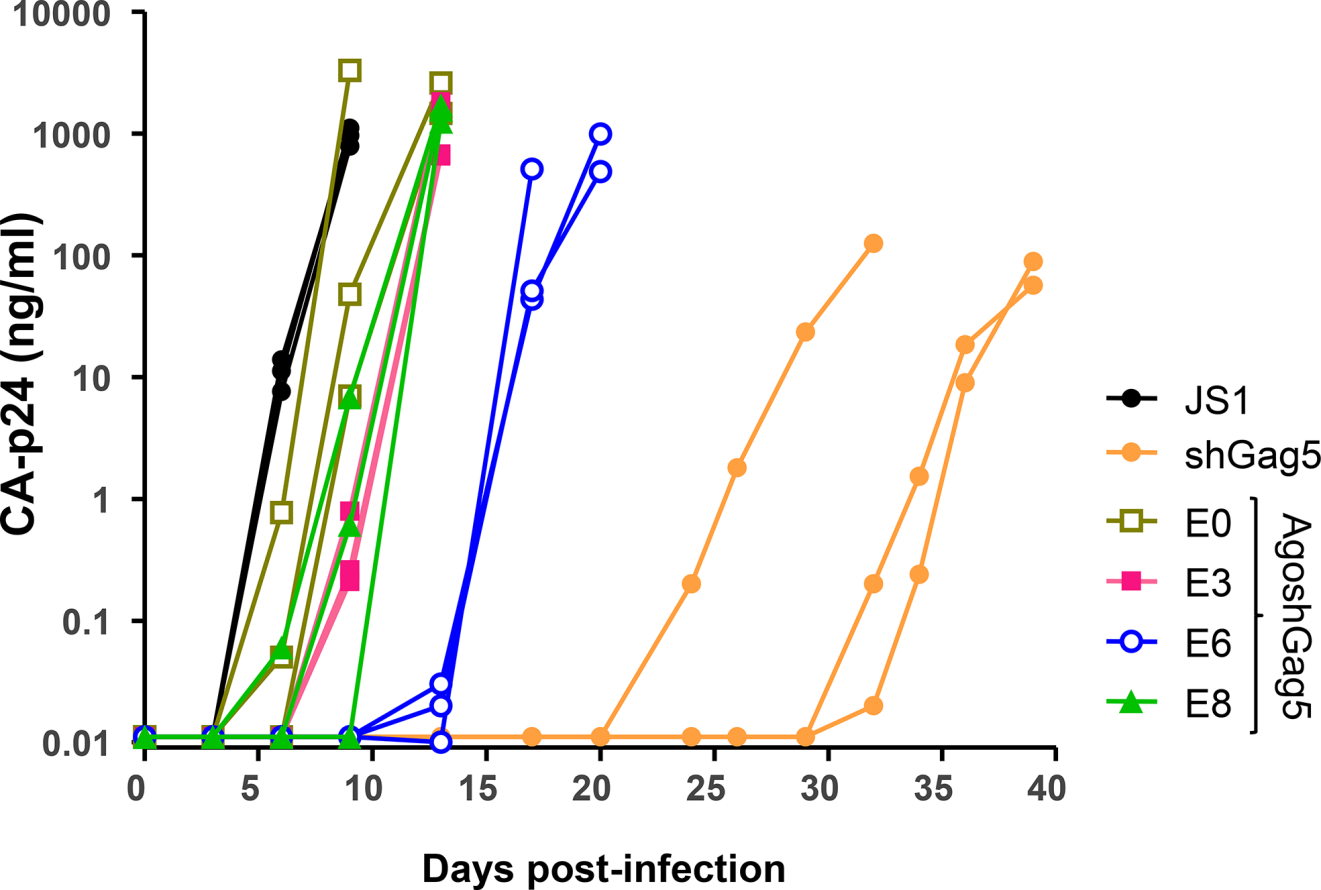
The impact of the AgoshGag5 variants on a spreading HIV-1 infection. Stably transduced SupT1 T cells expressing the AgoshGag5 variants or shGag5 were challenged with HIV-1 LAI at moi 0.02. Cells transduced with the empty lentiviral vector JS1 served as control. Virus replication was monitored by measuring CA-p24 in the supernatant for 39 days.

To further analyze the antiviral efficacy of the AgoshGag5 design, SupT1 T cells were transduced with a lentiviral vector (JS1) expressing the AgoshGag5 variants or shGag5. GFP-positive cells were sorted and subsequently infected with HIV-1 LAI at moi of 0.02. Cells transduced with the empty JS1 vector were used as negative control. We performed three independent infections for each cell line. Virus replication was followed for 39 days by monitoring the CA-p24 level in the culture supernatant (Fig 8). Cells transduced with the empty JS1 vector showed syncytia formation and virus replication at day 6. Virus replication was delayed for only a few days in the presence of AgoshRNAs E0, E3 and E8, but moderate inhibition of virus replication was observed for cells expressing E6, with no signs of virus replication up to 17 days. A more durable inhibition was observed for the positive control shGag5, with no signs of virus replication up to 32 days.

We tested for the generation of resistant HIV-1 variants by passage of the cell-free virus on restricted SupT1 cells. Subsequently, the genotype of the 19-nt target sequence and the flanking regions was determined by population sequencing. Point mutations within the target (A3G, 2xA6G) were detected for the shGag5-resistant cultures, but only wild-type sequences were observed for the AgoshGag5 cultures (E0, E3, E6 and E8). It is likely that no resistance was observed because the AgoshRNAs do not exert sufficient selection pressure on HIV-1. We previously described such break-through replication of wild-type virus in case of sub-optimal inhibitory activity [33].

### Destabilization of the AgoshRNA design by G-U base pairs

**Fig 9:**
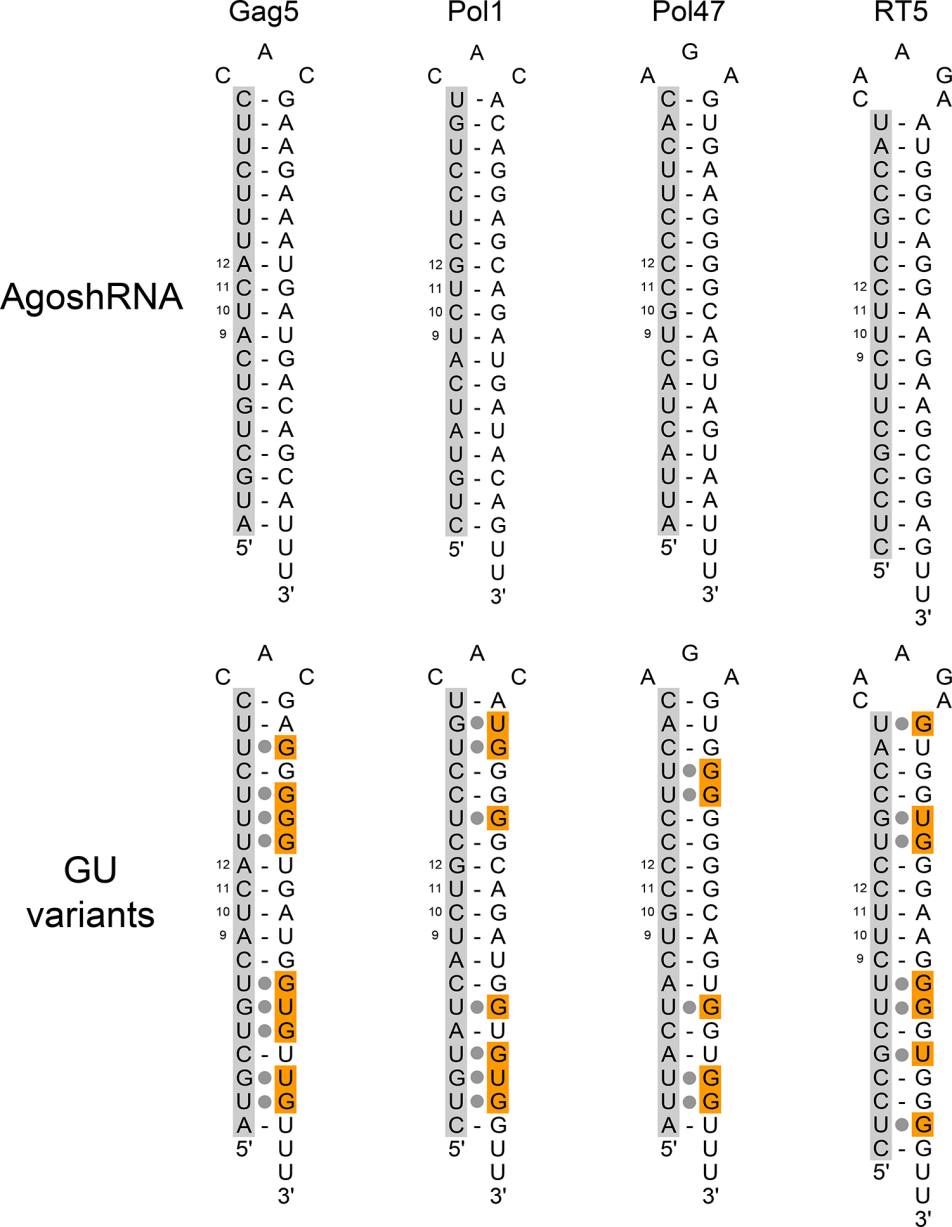
Modified AgoshRNAs with weakening GU basepairs. AgoshRNAs were modified by incorporation of G-U bp. G-U or U-G bp were introduced by mutation of the 3’ side of the stem (marked in orange), thus not affecting the 5’ side-encoded AgoshRNA guide (marked in grey). The mutations were avoided in the area where Ago2 cleaves (between bp 10 and 11). The thermodynamic stability (ΔG in kcal/mole) of the hairpins was determined using the Mfold program and indicated in Table 1.

Ago2-mediated processing of an AgoshRNA hairpin generates a single guide strand that could maintain basepairing in the upper hairpin domain. The 3’-end processing product is very short, but could also stick to the guide. These intramolecular and intermolecular basepairing interactions, respectively, may potentially block the accessibility of the AgoshRNA guide strand for its matching mRNA target and thus limit its activity. We thus wondered whether destabilization of the hairpin stem could improve the AgoshRNA activity. To do so, regular Watson and Crick bp in the four AgoshRNA inhibitors (Gag5, Pol1, Pol47 and RT5) were replaced by relatively weak G-U or U-G pairs. Mutations were introduced exclusively on the 3’ side of the duplex in order not to change the guide strand encoded on the 5’ side. We also avoided the central stem region around bp 10 and 11 where Ago2-cleavage occurs. Each U-A was changed to U-G and G-C was converted to G-U to destabilize the hairpin. This strategy results in 5 bp changes for Pol47, 7 for Pol1 and RT5 and 9 changes for Gag5 (Fig 9). The impact on the predicted thermodynamic stability of the hairpin is listed in Table 1. Stability decreases range from 2.0 kcal/mole for Pol47 to a mere 8.6 kcal/mole for Pol1. We also analyzed the effect for the top and bottom halves of the hairpin, mimicking the intramolecular and intermolecular interactions possible for the processed AgoshRNA strand that were introduced above. The largest top destabilization was apparent for Pol1 (4.4 kcal/mole) and a profound bottom destabilization of 7.2 kcal/mole was predicted for Gag5. We then measured the knockdown activity of these “GU weakened” AgoshRNA variants. No increased activity was scored for any of the GU variants compared to the original hairpins. In fact, the highly active AgoshGag5 and moderately active AgoshPol1 molecules lost nearly all activity (Table 1). Thus, GU introduction does not improve, but rather destroy the AgoshRNA activity, although one cannot exclude the possibility that precise introductions at certain positions of the hairpin will be beneficial for processing or activity.

**Table 1:** Stability and potency of the hairpins.

name	Complete hairpin	Top half	Bottom half	Luciferase knockdown	
	ΔG (kcal/mole)			sense	antisense
AgoshGag5	-27.7	-7.2	-12.8	+++	-
GU-AgoshGag5	-19.8	-6.5	-5.6	-	-
AgoshPol1	-33.3	-13.4	-12.8	+	-
GU-AgoshPol1	-24.7	-9.0	-8.6	-	-
AgoshPol47	-32.2	-14.1	-10.4	-	-
GU-AgoshPol47	-30.2	-12.8	-9.7	-	-
AgoshRT5	-37.0	-14.1	-16.3	+	-
GU-AgoshRT5	-29.4	-11.2	-12.2	+	-

Inhibition;-, no; +, 20–40%; ++, 40–70%; +++, 70–100%.

### Evaluation of AgoshRNA toxicity in human T cells

The main advantage of the AgoshRNA over the regular shRNA design is that this novel hairpin design reduces the chance of inducing unintended off-target and cytotoxic effects by the absence of a passenger strand, thus increasing their safety profile and therapeutic value. To test whether this is indeed the case, we transduced SupT1 cells with lentiviral constructs expressing AgoshRNAs against Gag (E0, E3, E6 and E8). As negative control, we included the empty lentiviral vector JS1 and the lentiviral vector encoding the H1 RNA polymerase III promoter. As positive control, we included shRT-B, which has previously been shown to trigger reduced cell growth [34]. The transduction was performed using two multiplicities-of-infection (moi): 0.225 and 0.45. The transduced cells express the shRNA and GFP reporter gene; the untransduced cells form the internal controls in these cultures. To determine the negative effect of AgoshRNA or shRNA expression on the growth of transduced cells, the percentage of GFP+ cells was monitored upon passage of these co-cultures for 50 days (Table 2). Like reported before [35], we observed a significant reduction in the percentage of GFP+ cells in the shGag transduction, comparable to that seen for the “toxic” shRT-B molecule. In contrast, none of the AgoshRNA-transduced cultures showed such a profound impairment of cell growth, although a slightly increased reduction in the percentage of GFP+ cells was measured compared to the negative control groups (JS1 and H1). Perhaps one would have expected some toxicity for the E8 Gag5 construct because it regains the ability to be processed as a shRNA by Dicer due to increased stem length. This would produce the shRNA-passenger strand that is the likely candidate for inducing off-target effects and toxicity. Indeed, E8 Gag5 produces the same amount of this passenger strand as shGag5 (Fig 6, lower left panel). However, the E8 passenger strand will be extended by 2 nt in the 5’ seed region due to the increased stem length. This is likely to affect the target-specificity and may thus have removed the toxic effect.

**Table 2:** Competitive cell growth (CCG) assay.

Lentiviral construct	MOI	Reduction in GFP+ cells (%)	MOI	Reduction in GFP+ cells (%)
JS1	0.225	1.52	0.45	0.86
H1	0.225	1.84	0.45	1.08
shRT-B	0.225	23.23	0.45	26.03
shGag	0.225	24.45	0.45	26.04
E0 Gag5	0.225	3.13	0.45	3.13
E3 Gag5	0.225	2.38	0.45	2.08
E6 Gag5	0.225	4.78	0.45	4.41
E8 Gag5	0.225	3.44	0.45	2.26

GFP expression of the cells was measured over 50 days.

## Discussion

The main advantage of the novel AgoshRNA reagents over the shRNA design is that only a single guide strand is generated, thereby reducing the chance of off-target effects on unrelated genes via the passenger strand. In addition, AgoshRNAs like miR-451 may be exclusively loaded in Ago2-containing RISC complexes [17], where it is processed and used as guide for mRNA cleavage. Avoiding Ago1, 3 and 4 will also reduce the chance of unwanted off-targeting via miRNA-like annealing to partially complementary target sites [36]. Furthermore, Ago2-mediated processing of AgoshRNAs predominantly yields a single mature product, whereas Dicer-processing of regular shRNAs creates imprecise ends [37, 38] (Harwig and Berkhout, unpublished results), which is related to the interaction with different Dicer-binding partners [39]. Thus, the AgoshRNA design seems ideal for therapeutic strategies.

In this study, we designed and tested 16 anti-HIV AgoshRNA designs by incorporating 4 previously proven shRNA-derived guide strands that efficiently target HIV-1 RNA [40] into the AgoshRNA backbone. Only 6 of these 16 AgoshRNAs were moderately to highly active in reporter silencing. This may seem a sobering score, but one should remember that it was also rather difficult to identify potent shRNAs. For instance, we screened a library of 86 shRNAs to identify 21 potent HIV-1 inhibitors [26]. Although RNAi tools are widely used, it remains difficult to design active shRNAs, in part because they do not follow the established siRNA rules [41–44]. In addition, HIV-1 may be a particularly constrained target mRNA because it is riddled with stable RNA structures that can effectively hinder RNAi attack [34, 45–47]. Even the most active anti-HIV AgoshRNA identified in this study (AgoshGag5), does not approach the activity observed for the matching shRNA that targets the same HIV-1 sequence.

On the positive side, while the shRNA induced guide and passenger strand activity, the AgoshRNA exclusively triggered guide-mediated knockdown of the luciferase reporter. The finding that these hairpins are exclusively loaded into Ago2-containing RISCs could reduce unwanted off-target effects by Ago1-3 mediated translational repression [17]. We addressed the toxicity induced by these small RNAs in a competitive cell growth assay. Whereas the shRNA had a profound impact on T cell growth, the cultures of the 4 AgohsGag5 variants showed only a minor effect. These combined findings illustrate the reduced off-targeting potential of AgoshRNAs over shRNAs, an important property for the future development of small therapeutic RNAs [48]. Nevertheless, AgoshRNA candidates should always be checked for adverse effects *in vitro* and *in vivo*.

The AgoshRNA variations tested in this study confirm the previously reported characteristics. Some of the modified AgoshRNAs returned to the shRNA pattern of Dicer-mediated processing upon extension of the stem length from 19 to 21 bp, which is consistent with our previous findings that a stem length of 17–19 bp is critical for Ago2-mediated processing [16]. We and others have recently demonstrated that the presence of a G-U bp in the top of the stem is beneficial for AgoshRNA activity [17, 22], but further G-U weakening did not improve the activity (this study).

In this study, we also tested several novel AgoshRNA modifications. First, we extended the mRNA-complementarity of the guide “over the loop” from 19 up to 27 bp, but measured no increased knockdown potency for such extended (E) variants. Second, we tried to optimize the AgoshRNA design by introduction of weak G-U bp along the stem, the rationale being that this may facilitate easier opening of the processed, but still basepaired molecule. No improved activity was scored for these GU variants. This modification even resulted in a loss of activity for a single hairpin, suggesting that the stem region may not fold correctly due to weakening.

For HIV therapeutics it remains of utmost importance to target highly conserved sequences to avoid viral escape and to target domains in the viral RNA genome that are accessible for the RNAi machinery [34, 49]. It seems important to screen more AgoshRNAs for anti-HIV activity and off-target effects. Although we know some of the rules for a successful AgoshRNA design (17–19 bp stem and small loop), there may be other important, yet undisclosed molecular determinants (Herrera-Carrillo and Berkhout, unpublished results). An alternative possibility is the use of miR-451 mimics, as this backbone may have been optimized during evolution for Ago2-mediated processing and silencing activity. For instance, an optimized miR-30 backbone was recently described with the evolutionarily conserved ACNNC element positioned 3’ of the stem [50]. This modification boosted pri-miRNA processing, resulting in increased levels of the mature RNA effector. Although pri-miRNA 30 processing is Drosha and Dicer dependent and AgoshRNAs are processed differently, these modifications might also be beneficial for the latter hairpins.

In sum, we demonstrate that active AgoshRNAs can be generated against HIV-1, although generally with reduced RNAi activity compared to the matching shRNA that targets the same viral sequence. We verified the absence of passenger strand activity for the AgoshRNA design, which seems a major bonus of over regular shRNAs. Indeed, a side-by-side comparison of a single AgoshRNA and the matching shRNA in sensitive cell culture based assays revealed much less toxicity for the former molecule. These combined data suggest that the future for AgoshRNA therapeutics may be bright, but further improvements are needed to increase their silencing activity.
